# Enhancement of PVDF Sensing Characteristics by Retooling the Near-Field Direct-Write Electrospinning System

**DOI:** 10.3390/s20174873

**Published:** 2020-08-28

**Authors:** Zheng-Yu Hoe, Chun-Chieh Chang, Jia-Jin Jason Chen, Chung-Kun Yen, Shao-Yu Wang, Yu-Hsuan Kao, Wei-Ming Li, Wen-Fan Chen, Cheng-Tang Pan

**Affiliations:** 1 Department of Biomedical Engineering, National Cheng Kung University, Tainan City 70101, Taiwan; jhoe@vghks.gov.tw (Z.-Y.H.); chenjj@mail.ncku.edu.tw (J.-J.J.C.); 2 Physical Medicine and Rehabilitation Department, Kaohsiung Veterans General Hospital, Kaohsiung 81362, Taiwan; 3 Department of Mechanical and Electro-Mechanical Engineering, National Sun Yat-sen University, Kaohsiung 80424, Taiwan; jay.chang@mem.nsysu.edu.tw (C.-C.C.); yen@mem.nsysu.edu.tw (C.-K.Y.); sywang@mem.nsysu.edu.tw (S.-Y.W.); kyh@mem.nsysu.edu.tw (Y.-H.K.); 4 Department of Urology, Kaohsiung Medical University Hospital, Kaohsiung 80756, Taiwan; wmli@kmu.edu.tw; 5 Department of Urology, School of Medicine, College of Medicine, Kaohsiung Medical University, Kaohsiung 80708, Taiwan; 6 Cohort Research Center, Kaohsiung Medical University, Kaohsiung 80708, Taiwan; 7 Department of Urology, Ministry of Health and Welfare Pingtung Hospital, Pingtung 90054, Taiwan; 8 Institute of Medical Science and Technology, National Sun Yat-sen University, Kaohsiung 80424, Taiwan

**Keywords:** piezoelectric fibers, servo motor controller, near-field electrospinning, polyvinylidene fluoride (PVDF), sensors

## Abstract

This research aimed to develop a direct-write near-field electrospinning system (DW-NFES) with three-axis positioning of controllable speed, torque and position to produce sizable and high-quality piezoelectric fibers for sensing purposes. Sensor devices with high electrical response signals were developed and tested. To achieve DW-NFES purpose, a servo motor controller was designed to develop a high response rate, accurate positioning, and stable mobile device through the calculation of bandwidth and system time delay. With this retooled system of DW-NFES, controllable and uniform size fibers in terms of diameters, stretching force, and interspaces can be obtained. Sensor devices can be made selectively without a complicated lithography process. The characteristics of this DW-NFES platform were featured by high response rate, accurate positioning, and stable movement to make fibers with high piezoelectric property. In this study, polyvinylidene fluoride (PVDF) was used to explore and enhance their sensing quality through the platform. The parametric study of the process factors on piezoelectric sensing signals mainly included the concentration of electrospinning PVDF solution, high voltage electric field, and collection speed. Finally, the surface morphology and piezoelectric properties of the as-electrospun PVDF fibers were examined by scanning electron microscopy (SEM) and characterized by electrical response measurement techniques. The results showed that the fiber spinning speed of the DW-NFES system could be increased to ~125 from ~20 mm/s and the accuracy precision was improved to ~1 from ~50 μm, compared to conventional step motor system. The fiber diameter reached ~10 μm, and the electrospinning pitch reached to as small as ~10 μm. The piezoelectric output voltage of the electrospun fibers was increased ~28.6% from ~97.2 to ~125 mV; the current was increased ~27.6% from ~163 to ~208 nA, suggesting that the piezoelectric signals can be enhanced significantly by using this retooled system. Finally, an external control module (Arduino-MAGE) was introduced to control the PVDF piezoelectric fiber sensors integrated as a sensing array. The behavior of long-term sedentary patients can be successfully detected by this module system to prevent the patients from the bedsores.

## 1. Introduction

In the modern and booming technology industry, as people are constantly pursuing products which are smaller, lighter, and more versatile, the demand for precision manufacturing technology is increasing. In the manufacturing process, motors are required to achieve high-speed and precise processing, which can be applied to high-tech industries such as computer numerical control (CNC) numeric control machines, semiconductor industry, micro/nano structures, and precision machinery. Through this technology, the polymer is separated from the application field of conventional materials, and becomes one of high-tech such as high-performance materials, biology, and optoelectronics. In recent years, intelligent fibers and fabrics made out of nanofibers have been increasingly valued by the market [1], the stability of equipment and mass production are indispensable. Therefore, to develop a controller which is suitable for a high-yield spinning machine becomes a significant subject.

The conventional electrospinning technology used high voltages to fabricate nanofibers [2]. The positive electrode was connected to the top of the metal needle, and the negative electrode (ground) was connected to the metal plate, a high-voltage electric field was formed in the electrospun region. When the electrospinning solution was on the top of the needle and balanced by the force of the high-voltage electric field and the surface tension of the droplet, the droplet formed a conical ridge, which was called the Taylor cone [3,4,5]. When the droplet continued to be stretched by the electric field till broken through the top of the Taylor cone, the top produced a long strip of liquid, and continuously fell on the collecting plate to form a micron-sized fiber. In the process of collecting fibers, the liquid column was turbulent and twisted. Therefore, the electrospun fiber did not go along with a fixed path, and there was no fixed directivity of collection, thus the collection method and stability became important issues.

The directionality of material collection is more significant in the use of piezoelectric materials. Polyvinylidene fluoride (PVDF) used in the study is a high molecular polymer. In 1969, Kawai et al. [6] found the excellent piezoelectric properties of PVDF after polarization and stretching under a high voltage electric field, its piezoelectric coefficient d_33_ is about −34 pC/N. PVDF contains crystal phases of α, β, γ, δ, ε [7], wherein β is the main power generation phase of piezoelectric materials, which electromechanical conversion efficiency is the highest among all phases. The γ phase is a mixture of the α phase and β phase, and piezoelectric characteristics of the γ phase are inferior to the β phase [8]. PVDF powder and film are generally composed of the α phase, and their dipoles are non-directional, consequently, the charges easily offset each other, resulting in lack of piezoelectric characteristics. As a result, it is necessary to convert PVDF into the β phase through a polarization process such as electrospinning [9,10,11]. The PVDF material was produced with high piezoelectric property in many polarization processes, such as annealing process for secondary crystallization, phase transitions during stretching, conformation transition behavior during melting process, and conformational behavior of polar polymer models under electric field [12,13]. The β-phase of PVDF with the permanent electric dipole perpendicular to the axis with corresponding strong and ferroelectric and piezoelectric charges were produced due to a trans-gauche-trans-gauche’ (TGTG’) atomic arrangement of a centrosymmetric unit cell, CH_2_, and perpendicularity of the dipoles and CF_2_ [14,15].

In the development of the application, PVDF fibers were widely manufactured in 2005 using the conventional electrospinning technology [16,17,18]. Since β-phase PVDF fibers were irregularly attached to the fiber collecting plate, the dipole moments canceled each other. Thus, the conventional PVDF fiber is free of piezoelectric characteristics. In 2006, Lin et al. proposed a near-field electrospinning (NFES) technology to successfully spin nanofibers in an orderly pattern by reducing the distance which was from 10 cm to 1 mm between the needle and the collecting plate [19,20,21]. Therefore, the path of electrospun fibers could be precisely controlled. However, spacing of electrospun fibers and limited fiber diameter were mainly confined by electrospinning equipment. As a result, to precisely adjust both solution supply device and collection device in NFES are significant factors affecting the quality of electrospun fibers. In view of the fact that automation was a new trend in modern times, precision products gain competitive advantages, thus precision micro-control play an essential role in modern control design.

This study developed a direct-write near-field electrospinning (DW-NFES) platform with three-axis positioning of controllable speed, torque, and position to produce uniform-sized and high-quality piezoelectric fibers for sensing purpose. A servo motor controller was designed to develop a high response rate, accurate positioning, and stable mobile device through the calculation of bandwidth and system time delay. The piezoelectric fibers were made using this retooled platform. A sensitive sensor device can be made selectively without the complicated lithography process. PVDF material was utilized to characterize its sensing quality by using this DW-NFES platform. The surface morphology and piezoelectric properties PVDF fibers were examined. By the method of high-order control to retool the DW-NFES system, the parallel electrode was combined with multiple pairs interdigitated electrodes and the repolarization method. The electrical signal output (voltage) of PVDF piezoelectric fiber sensors was improved. The sensing system directly read the signals without an amplifier circuit. The system would continuously output messages to the readers to remind and warn the caregiver whether or not the patients sit for a long time. Hence, this module system could potentially prevent patients from potential bedsores.

## 2. Research Methods

### 2.1. Electrospinning Process

**Figure 1:**
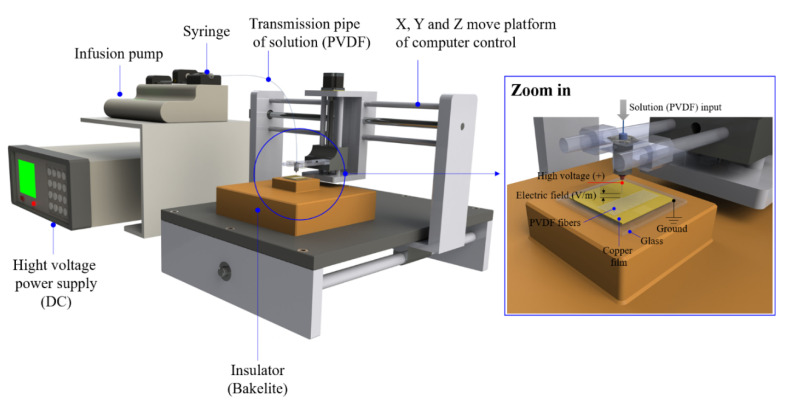
Developed a direct-write near-field electrospinning (DW-NFES) platform.

To improve the collection efficiency, the collected devices on the platform were driven by a motor. The speed and positioning accuracy were improved by replacing the conventional stepping motor with a servo motor with better performance in this research. The increase in transmission speed increased the production of fiber manufacturing. Fiber diameters could also be reduced. High positioning accuracy increased the number of fibers which were covered on the identical area and increased the fiber density. Differences in products were examined through electrical measurements and surface observations. Furthermore, through the exploration of the motor parameters, the quality of platform motion was improved including increment of response speed and decrease of the speed ripple in motion. The established platform is shown in Figure 1. For the PVDF piezoelectric fiber process, PVDF powder was prepared into two liquid solutions. The first solution consisted of acetone to dissolve PVDF powder (M.W. about 534,000 g/mole), and the second solution consisted of dimethyl sulfoxide with an anionic surfactant. Then, two solutions were completed by mixing and stirring at a speed of ~500 rpm for 60 min. Finally, the PVDF solution was prepared into uniform fibers with high piezoelectric properties by the NFES technology. The electrical signals of piezoelectric fibers were able to be collected through electrodes.

**Figure 2:**
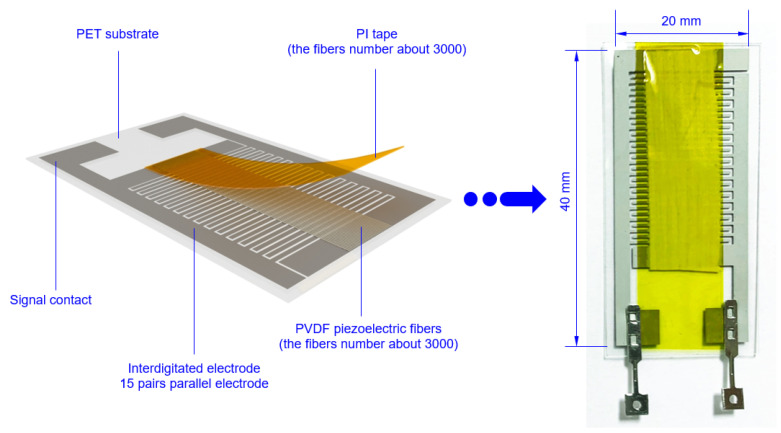
Schematic diagram of the interdigitated (IDT) electrode.

**Figure 3:**
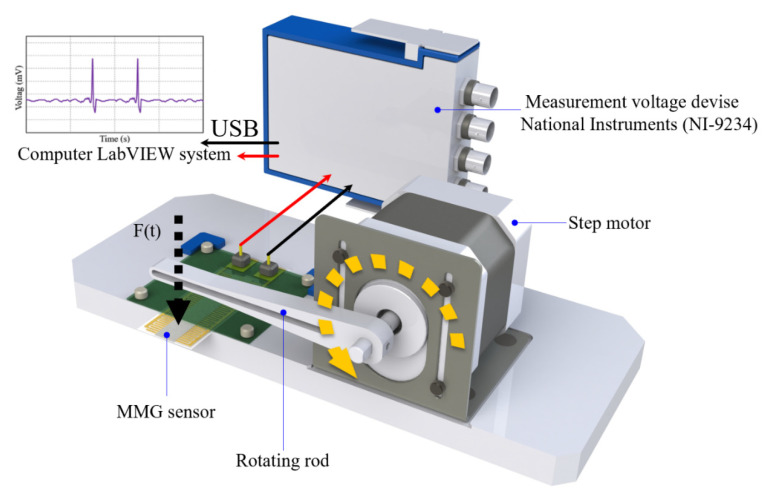
Electrical measuring equipment to capture Polyvinylidene fluoride (PVDF) piezoelectric fibers sensor signals.

In order to enhance the signal-collecting efficiency, a planar interdigitated (IDT) electrode was designed to capture the signals from the PVDF fibers in Figure 2. The IDT electrode width is 0.6 mm, the distance between the two electrodes is 0.2 mm, and a total of 15 pairs of electrodes. The material of IDT electrode was PAX-767-2 (Advanced Electronic Materials Inc., Tainan, Taiwan) with excellent electrical and physical properties. It is a halogen-free conductive silver paste which was supplied by Advanced Electronic Materials Inc. (ITK), as shown in Table 1. Then, the equal-area electrospinning (length 40 mm, width 8 mm) was attached to the IDT electrode sheet and polarized by 1400 V high voltage for 60 min. Then, the energy extraction device was installed to measure the voltage, current, and strain signals in the d_33_ piezoelectric mode, as shown in Figure 3. Finally, the surface morphology and piezoelectric properties of the as-electrospun PVDF fibers were examined by a SEM.

### 2.2. Motor Comparison and Servo Controller Design

The motor selected for precise control is a stepper motor and servo motor. The servo motor adopts the ML1513d model, and the stepper motor adopts 57BYGH56-401A. The main difference between the two models is the control of the open-loop and the closed-loop. In positioning, the stepping motor used the step angle as the minimum positioning accuracy where the step angle was 1.8°. Therefore, the control accuracy reached 1.8° only, and the linear displacement accuracy was 0.05 mm through the screw conversion. The servo motor was positioned by the encoder. The encoder is an optical encoder with 2500 pulses on one side, 10,000 resolutions on quadruple frequency, and linear displacement accuracy of 0.5 μm with a screw. On the other hand, in the stepping motor, as the speed increased, the torque was reduced, and the out-of-step condition occurred. However, the servo motor ensured constant power and constant torque. In order to improve the process technology, the traditional stepping motor was replaced with a servo motor. A comparison of these two motors is shown in Table 2. After comparison, the design of the controller was found to be the key technology of the servo motor.

**Figure 4:**
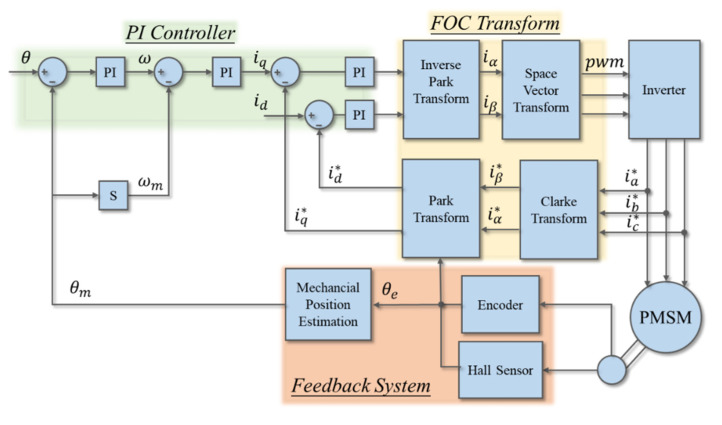
Servo motor drive flow chart.

The drive flow of the servo motor included the PI controller design, the field-oriented control (FOC) conversion, and the feedback system, as shown in Figure 4. The FOC conversion converted the three-phase AC control into a simpler DC control to provide the PI controller design. In the feedback system, the hall sensor and the encoder could reach the accuracy of 1/10,000 ramp. Especially, a good PI controller parameter required to be accurately and quickly moved to the target angle. For electrospinning platform systems requiring high precision positioning, the PI controller design was an important key. Therefore, this paper proposed two PI design methods to compare, where one was the traditional extreme zero elimination method, and the other was the optimal bandwidth symmetry method.

**Figure 5:**
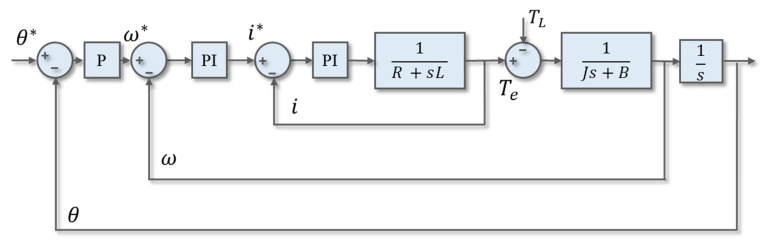
Servo motor control loop.

In the traditional design method, the pole-zero cancellation method was designed in the motor three-loop transfer function, as shown in Figure 5. In the current loop design, 1/20 of the PWM switching frequency was used as the design criterion. *G_i_*(s) was an open loop current transfer function which consisted of a controller and a motor current model. The pole-zero elimination method was applied to (*s* + *k_i_I_/k_p_I_*) = (*s* + *R_q_*/*L_q_*) and was able to be obtained in Equation (1):

(1) $$G_{i}\left(s\right)=\frac{K_{p\_I}\left(s+K_{i\_I}/K_{p\_I}\right)}{s}\cdot \frac{1}{L\left(s+R/L\right)}$$

where *K_p___I_* is the proportional gain of the current loop, *K_i_I_* is the integral gain of the current loop, *R_q_* is the motor resistance, and *L_q_* is the motor inductance.

Thus, the closed loop transfer function was shown in Equation (2), the proportional and integral gains of the current controller were shown in Equations (3) and (4):

(2) $$G_{ic}\left(s\right)=\frac{\frac{K_{p\_I}}{L}}{s+\frac{K_{p\_I}}{L}}=\frac{\omega_{i}}{s+\omega_{i}}$$

(3) $$K_{p\_I}=\omega_{i}\cdot L$$

(4) $$K_{i\_I}=\omega_{i}\cdot R$$

where *ω_i_* was the bandwidth of current loop.

The same concept can be used to represent that *G_n_*(s) was an open loop speed transfer function which consisted of a controller and a motor mechanical model, as shown in Equation (5):

(5) $$G_{n}\left(s\right)=\frac{K_{p\_N}\left(s+K_{i\_N}/K_{p\_N}\right)}{s}\cdot \frac{1}{J\left(s+B/J\right)}=\frac{K_{p\_N}}{J\cdot s}$$

where *K_p_*_*_N_* is the proportional gain of the speed loop, where *K_i_*_*_N_* is the integral gain of the speed loop, J is the motor inertia, and B is the friction coefficient.

Thus, the closed loop transfer function became Equation (2), the proportional and integral gains of speed controller were obtained:

(6) $$K_{p\_N}=\omega_{n}\cdot J$$

(7) $$K_{p\_N}=\omega_{n}\cdot B$$

where *ω_n_* is the bandwidth of speed loop and is set to 0.1*ω_i_*.

For the position controller design, *G_p_* is an open loop transfer function:

(8) $$G_{p}(s)=\frac{K_{p\_P}}{s}$$

where *K_p__P* is the proportional gain of the position loop, *ω_p_* is the bandwidth of position loop and is set to 0.1*ω_n_*. Therefore, the proportional gain of position controller is obtained:

(9) $$K_{p\_P}=\omega_{p}$$

**Figure 6:**
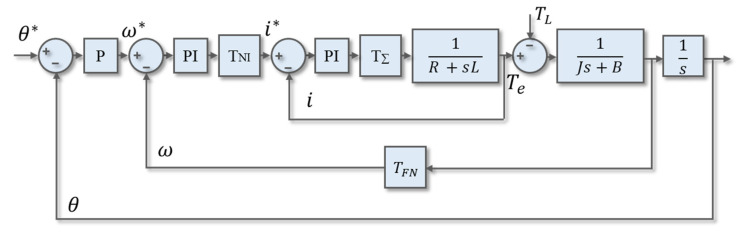
Servo motor control loop with delay time.

In this study, a new parameter design method was proposed as shown in Figure 6. This method designed each loop parameter by considering the delay time to achieve a faster and more stable response. In each loop, the bandwidth of its transfer function was designed, called the high bandwidth design method.

The open-loop transfer function of the current loop *G_OI_* (s) is represented by Equation (10). The current loop consisted of the PI controller, system delay, the motor electrical model, and PI controller:

(10) $$G_{OI}(s)\;=\;\frac{K_{PI}\cdot (1+s\cdot T_{NI})}{s}\cdot \frac{1}{R\cdot (1+\frac{s\cdot L}{R})}\cdot e^{-sT_{\Sigma }}$$

where $e^{-sT_{\Sigma }}$ is the time delay and KPI and TNI stand for the PI controller gain and time constant in the speed loop.

Controller design was based on the pole-zero elimination method. The ratio of current controller gains could be derived as *T_NI_* = *R/L*. Therefore, *G_ol_*(s) were rewritten as shown in Equation (11):

(11) $$G_{OI}(s)=\frac{K_{PI}}{Ls}e^{-sT_{\Sigma }}=\frac{\gamma }{T_{\Sigma }s}e^{-sT_{\Sigma }}$$

Setting phase gain γ and design the parameter *K_PI_*:

(12) $$\gamma =\frac{K_{PI}T_{\Sigma }}{L}$$

The phase margin *Φ_R_* was derived obtained:

(13) $$\emptyset_{R}\;=90{}^{\circ}-180{}^{\circ}\frac{180{}^{\circ}}{\pi }\gamma$$

Therefore, the relationship between the controller parameters and the phase margin were obtained from Equation (13). In order to attain the parameters of the formula, the phase margin was defined as 60° [22].

The open-loop transfer function of the speed loop *G_ON_*(s) is represented by Equation (14), which consisted of a PI controller, speed loop delay, and a motor mechanical model:

(14) $$G_{ON}(s)\;\approx \;K_{PN}〔1+\frac{1}{s\cdot T_{NN}}〕\cdot \frac{1}{sJ}\cdot \frac{1}{1+s\cdot T_{\Sigma N}}=\;\frac{K_{PN}(1+s\cdot T_{NN})}{s^{2}J(1+s\cdot T_{\Sigma N})}$$

where *K_PN_* and *T_NN_* are the PI controller gain and time constant in the speed loop, respectively. Speed loop delay ($T_{\Sigma N}$) consists of the speed filter delay and speed calculation delay.

The design parameter *α* was introduced in the symmetric optimal method [23]. The concept of symmetrical optimum selects the crossover frequency at the geometric mean of the two corner frequencies. The *α* value was set as 2 to 4 in normal. The controller gain (*K_PN_*) and speed controller time constants (*T_NN_*) were shown in Equations (15) and (16), respectively. The closed loop transfer function *G_CN_(s)* was shown in Equation (17):

(15) $$K_{PN}=\frac{J}{\alpha T_{\Sigma N}}$$

(16) $$T_{NN}=\alpha^{2}T_{\Sigma N}$$

(17) $$G_{CN}(s)=\frac{1+sT_{NN}}{1+sT_{NN}+s^{2}\frac{T_{NN}J}{K_{PN}}+s^{3}\frac{T_{NN}T_{\Sigma N}J}{K_{PN}}}$$

To eliminate the overshoot due to the numerator of speed loop, a low pass filter (*G_f_*(s)) was used to eliminate the zero point of the speed loop, as shown Equation (18). The time constant of low pass filter *T_σ_* was set to *T_NN_*. Therefore, the modified speed closed loop transfer function were shown in Equation (19):

(18) $$G_{f}\left(s\right)=\frac{1}{1+s\cdot T_{\sigma }}$$

(19) $$G_{CN}{}^{\prime }\left(s\right)=\frac{1}{s^{3}\cdot (\frac{JT_{NN}T_{\Sigma N}}{K_{PN}})+s^{2}\cdot (\frac{JT_{NN}}{K_{PN}})+s\cdot T_{NN}+1}$$

For the same concept to the position loop, the magnitude of *G_CN_’*(s) was a unit. Equation (20) was derived as the closed loop transfer function *G_CP_*(s). The position control gain (*K_PP_*) was obtained by position controller and 1/*s* to eliminate the speed overshoot, as shown in Equation (21):

(20) $$G_{CP}(s)=\frac{K_{PP}}{s+K_{PP}}$$

(21) $$K_{PP}=\frac{1}{T_{NN}}$$

### 2.3. Design of Parallel Electrode and Interdigitated Electrode

**Figure 7:**
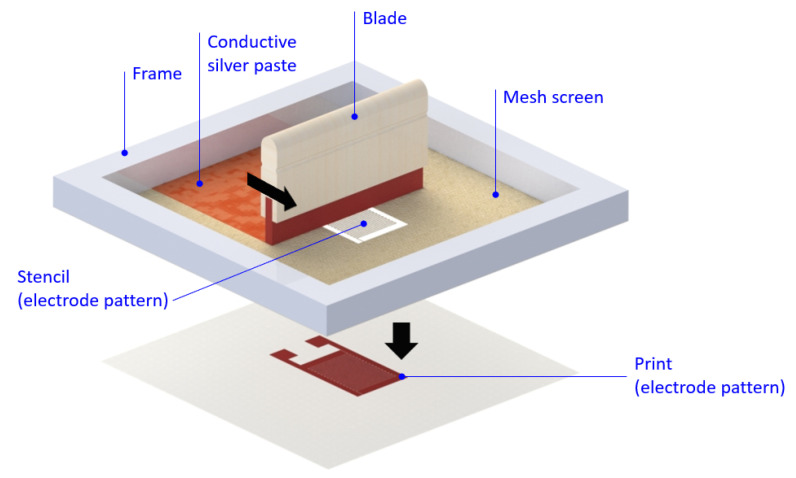
The screen printing process.

The PVDF piezoelectric fibers were polarized by a high voltage electric field and tensile stress during electrospinning. Due to the insufficient polarization time, it would cause low polarization of PVDF piezoelectric fiber. Therefore, through the parallel electrodes and the interdigitated electrodes of different pole pairs, repolarization was applied. The signal of the PVDF piezoelectric fiber sensor was improved through the repolarization process. The electrode (parallel electrode and interdigitated electrode) was manufactured by screen printing. A stencil was used to transfer the conductive silver paste (Advanced Electronic Materials Inc., PAX-767-2, Tainan, Taiwan) onto a PET (Perm Top Co., Ltd., Taipei, Taiwan) substrate (thickness is 200 μm) in the screen printing process. Firstly, the blade was moved across the screen to fill the open mesh apertures with conductive silver paste, a reverse stroke then caused the screen to contact the substrate momentarily along a line of contact. This causes the conductive silver paste to wet the substrate and be pulled out of the mesh apertures as the screen springs back after the blade has passed. Then, the silver electrodes on the PET substrate was printed. Finally, the electrode of printing was cured at a temperature of ~130 °C for 30 min, the screen printing process was shown in Figure 7.

**Figure 8:**
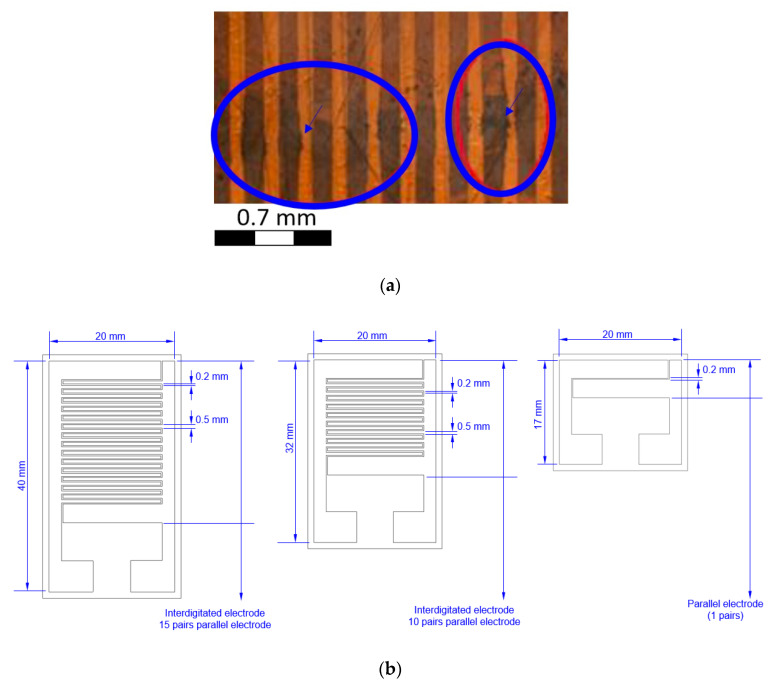

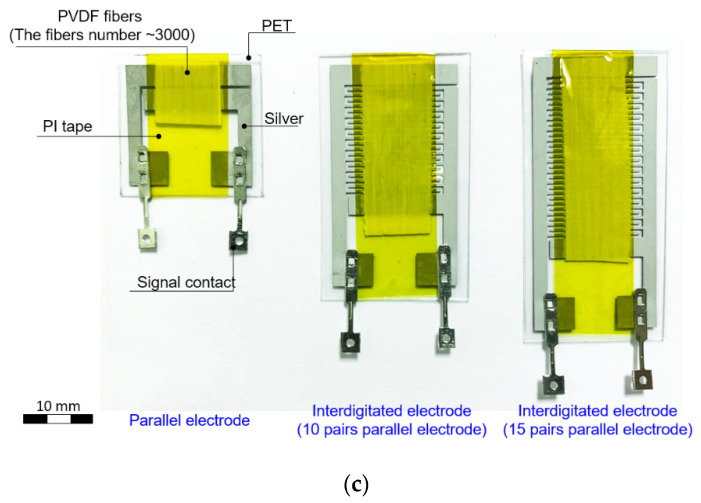
(**a**) The interdigitated electrode wire gap less than 0.15 mm was destroyed with high voltage; (**b**) illustration of interdigitated electrodes with different pole pairs; (**c**) Parallel electrodes and interdigitated electrodes with different pole pairs of 0.2 mm gap.

During the experiment, it was found that the electrode gaps exceeding 0.15 mm was lead to an insignificant repolarization effect in the PVDF fibers. When the gap is less than 0.05 mm, the electric current would destroy the interdigitated electrode structure, as shown Figure 8a. Therefore, the gap between each pair of electrodes was set at 0.2 mm. The PVDF piezoelectric fibers (diameter is 10 mm, length increases according to different pole pairs) was attached on parallel electrodes and interdigitated electrodes of different pole pairs, as shown in Figure 8b. Finally, the PVDF piezoelectric fiber sensor was completed, as shown in Figure 8c.

### 2.4. Repolarization Process

**Figure 9:**
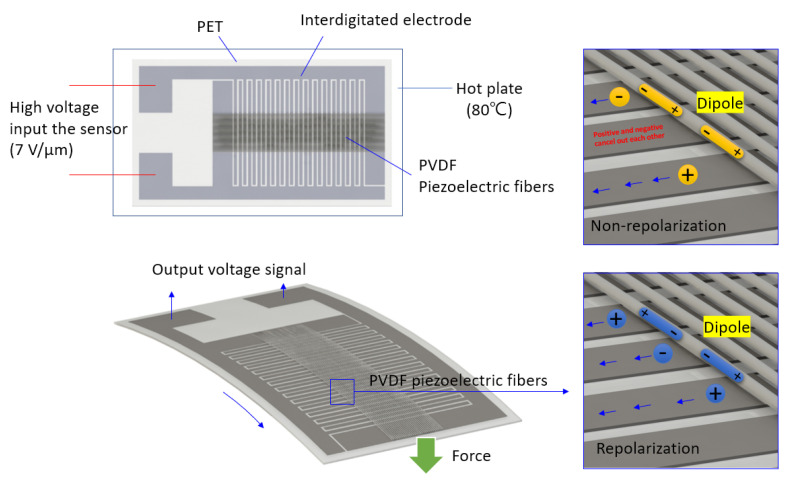
Illustration of PVDF piezoelectric fibers (area: 28 × 10 mm^2^, about 3000 fibers) that were placed on various flexible (PET) interdigitated electrodes.

The electric field and temperature were the important factor in the piezoelectric β-crystalline phase formation, the repolarization process added a high-voltage field to align dipoles in sequence and heated the PVDF fibers to increase the activity of dipoles momentum. Figure 9 shows the PVDF piezoelectric fibers (area: 28 × 10 mm^2^, about 3000 fibers) that were placed on various flexible (PET) interdigitated electrodes. Through the repolarization process with 7–8 V/μm (~1400 V) at 80 °C for 2 h, the fibers generated a regional dipole moment corresponding to the electrode pairs. For the measurement process, compression and tension were exerted on the piezoelectric fibers to obtain the potential voltage. The bending force resulted in mechanical strain, which distributed along the fibers and then converted to alternating voltage and current through the piezoelectric d_33_ mode.

## 3. Results and Discussion

### 3.1. Response Analysis of Servo Motor Controller Design

**Figure 10:**
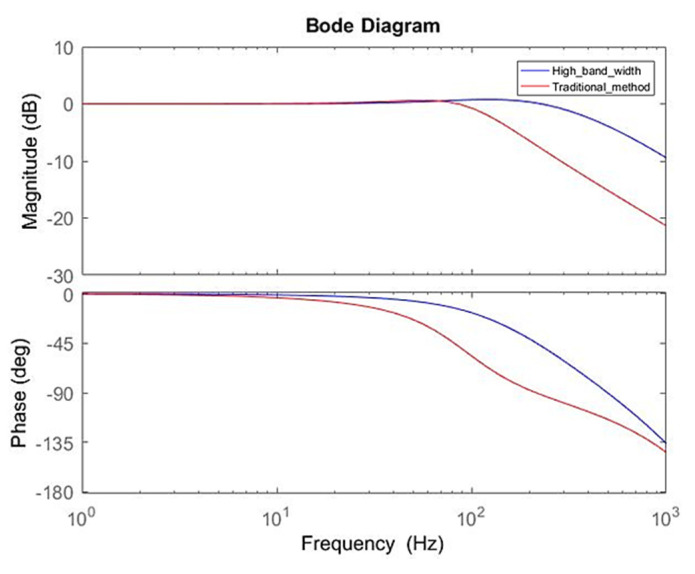
Bode diagram of current loop.

**Figure 11:**
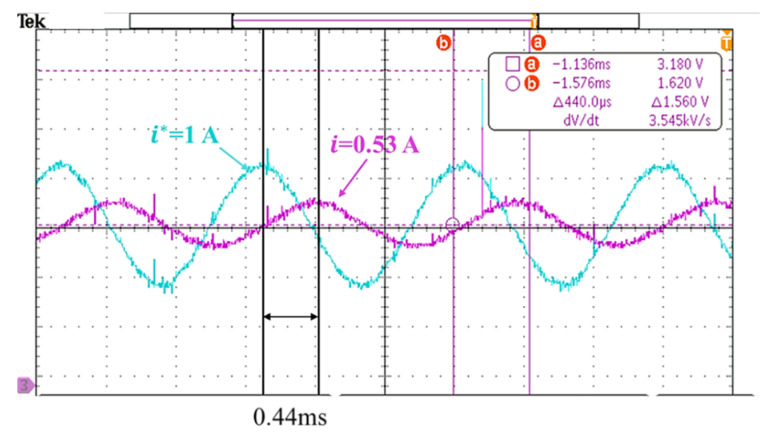
Current loop frequency response verification.

**Figure 12:**
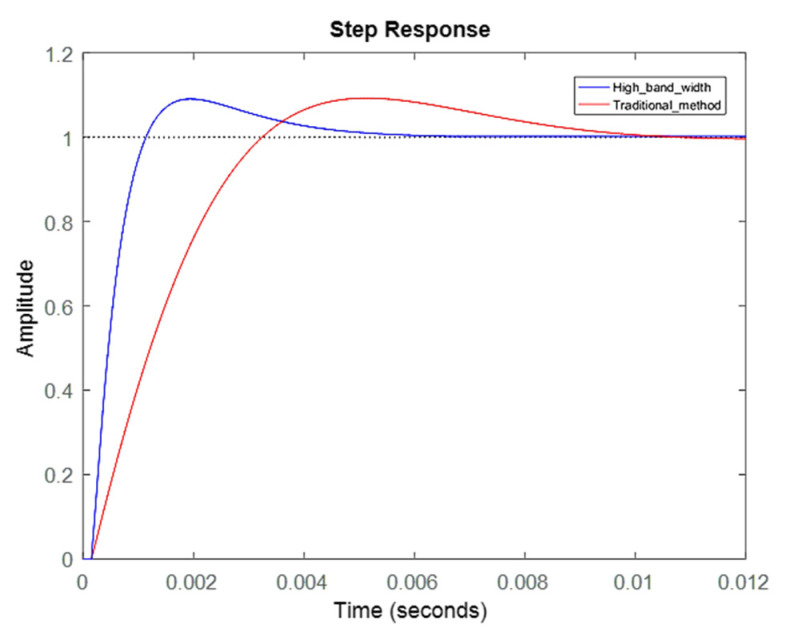
Current loop step response.

The effects of different controller parameters on the motor were investigated. Through the optimization of parameters, the operational accuracy of the motor was improved, and the response was achieved more rapidly with a superior yield. The performance of the motor could be expressed by the frequency response and the time domain response. The simulation results on the bode diagram showed that the current loop bandwidth was elevated from ~137 to ~437 Hz, as shown in Figure 10. In order to verify the simulation results, a current loop experiment was carried out through the frequency of the 90° phase lag in the bode diagram in Figure 10. In the measurement of the current loop, a sine wave command was inputted with an amplitude of 1 A at 500 Hz. The follow-up response was ~0.53 A, which was a drop of ~5.5 dB. As shown in Figure 11, the correctness of the simulation was demonstrated. In Figure 12, the simulation of the time-domain response was represented by a step response. The blue line is the curve of the high bandwidth design, and the red line is the curve of the traditional design. In the case of the same overshoot, there was an earlier response in simulation. From Table 3, the rise time was reduced from ~2.3 to ~0.69 ms.

**Figure 13:**
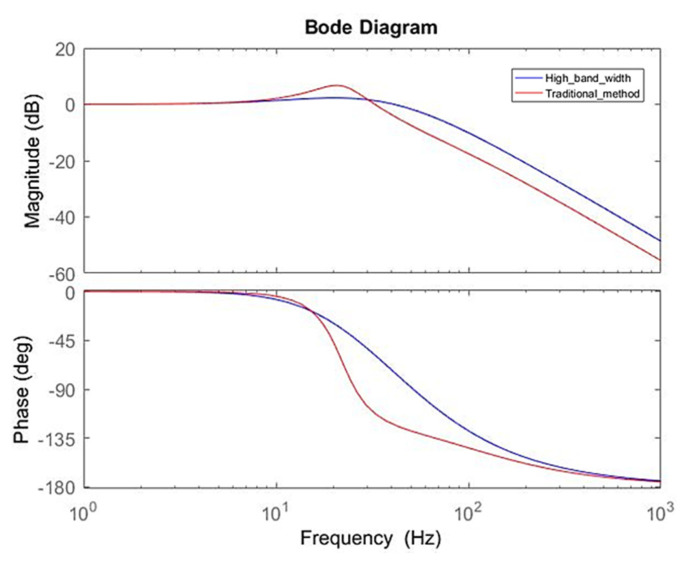
Bode diagram of speed loop.

**Figure 14:**
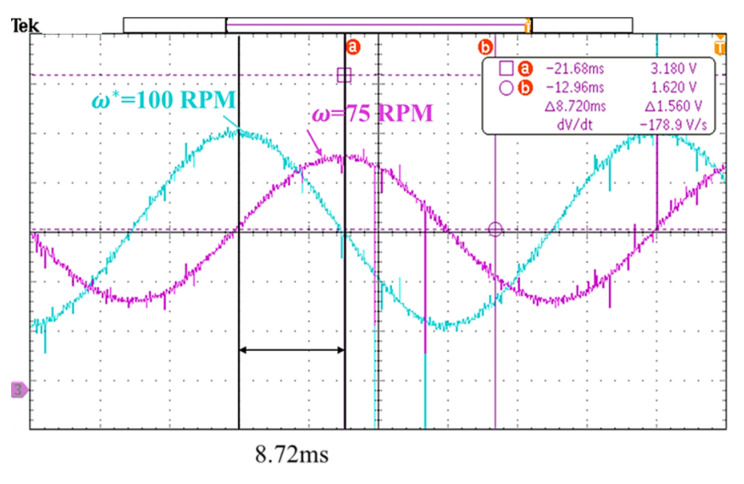
Speed loop frequency response verification.

**Figure 15:**
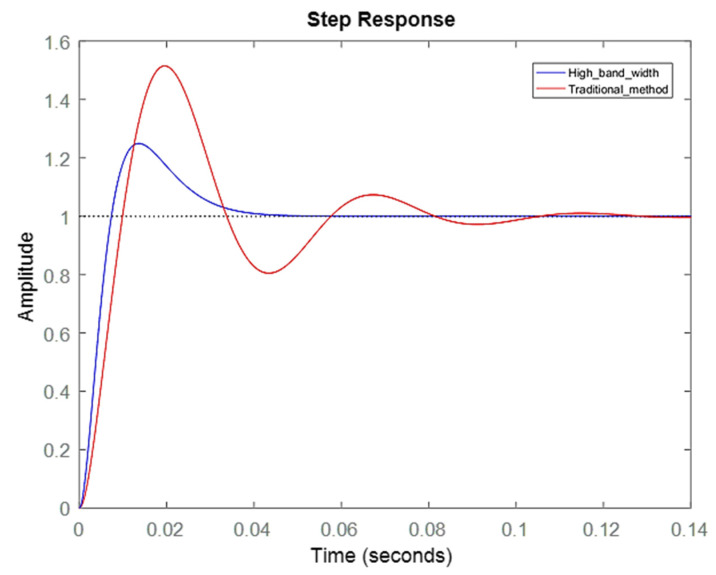
Speed loop step response.

The same simulation and verification method were used on the speed loop. In Figure 13, the bandwidth was increased from ~38 to ~57 Hz and the bode diagram in high bandwidth method (blue line) became smoother. In the speed loop experiment, a sine wave command was inputted with an amplitude of ~100 RPM at ~50 Hz. The follow-up response was ~75 RPM, which was a drop of ~2.5 dB, as shown in Figure 14. As a result, the correctness of the simulation was demonstrated. In Figure 15, the step response at speed was directly related to the movement of the platform. There was a large speed ripple in the simulation of the traditional design method. Faster and more stable responses in the high bandwidth design method was obtained. From Table 4, the rise time decreased from ~6.9 to ~2.1 ms, and the overshoot decreased from ~51.5% to ~24.9%.

### 3.2. Comparison of the Performances between Stepper Motors and Servo Ones

**Figure 16:**
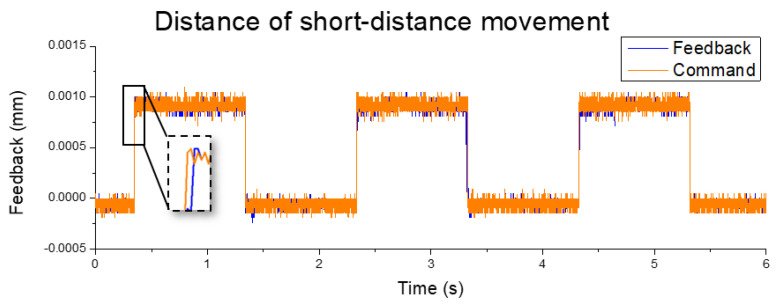
Position feedback measurement.

**Figure 17:**
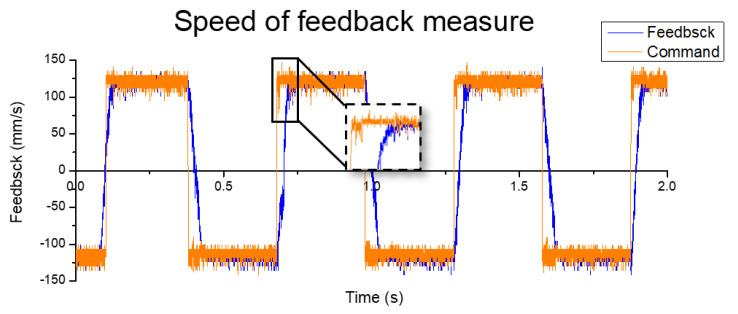
Speed feedback measurement.

The stepper motor was used in the original platform as an open-loop control system. The out-of-step and inaccurate position control occurred under the high-speed condition. As a result, the operative speed must be maintained below ~20 mm/s, and the accuracy was limited by the step angle of 1.8°. Once converting to linear movement, the minimum moving distance was 50 μm in accuracy. In this study, the step motor was replaced with a proposed servo motor. In a short-distance movement test with the symmetric optimal method, the displacement accuracy reached 1 μm and the response time was 4 ms, as shown in Figure 16. With a speed change from −125 to 125 mm/s, the response time was 34 ms, as shown in Figure 17. Finally, comparison of the results of the two motors is shown in Table 5.

### 3.3. Comparison of the As-Electrospun Fibers with Ones by Different Motors

**Figure 18:**
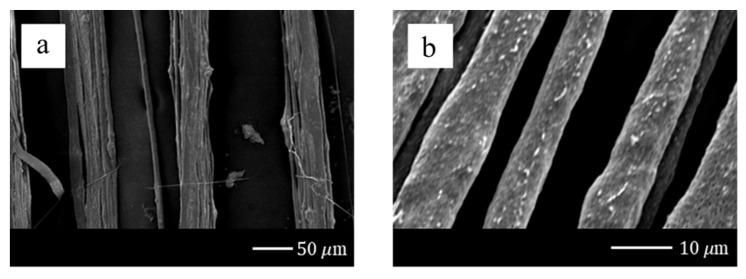
Diameter analysis of electrospinning fiber by (**a**) the stepper motor, (**b**) the servo motor.

**Figure 19:**
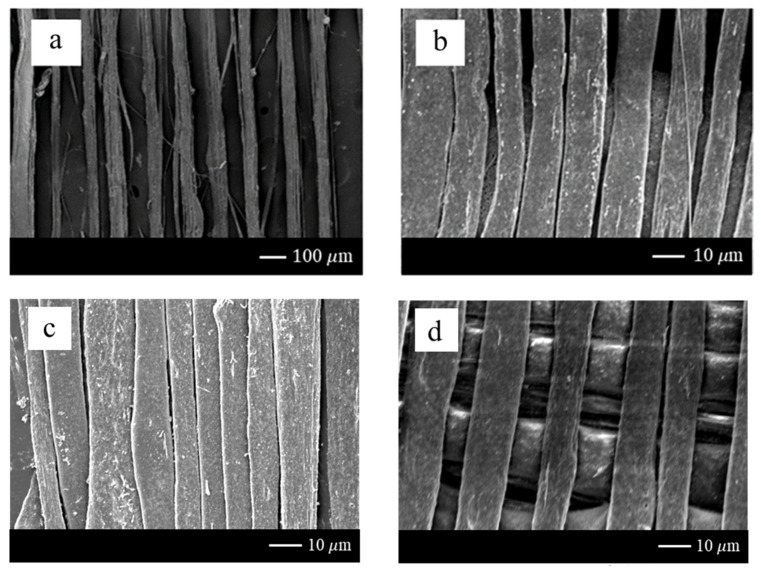
Density analysis of electrospinning: (**a**) 50 μm of the pitch of the stepping motor; (**b**) 10 μm of the pitch of the servo motor; (**c**) 7 μm of the pitch of the servo motor; (**d**) cross-laminated fiber of the servo motor.

The parameters of the PVDF fibers fabrication was that the diameter of the syringe was 0.33 mm, the concentration of the PVDF solution was 18 wt%, the voltage was set as 12 kV, and the electric field was defined to 1.2 × 10^7^ V/m. The infusion pump fed the electrospinning solution at 0.01 mL/h. In Figure 18, the maximum speed of the stepper motor was 30 mm/s, the diameter of the electrospun fiber was ~30 μm. The speed of the DW-NFES developed in this project reached ~125 mm/s. The average diameter of electrospun fibers was ~10 μm. These results demonstrated that the higher the speed, the finer the fiber can be formed. In addition, it can be found that the surface of the electrospun fiber was quite intact due to the high stability of the servo motor. The density analysis is shown in Figure 19. The minimum electrospinning pitch of the stepping motor was ~50 μm, as shown in Figure 19a. After replacing with the servo motor, the minimum electrospinning pitch of the servo motor could be up to ~1 μm. However, when the moving pitch was smaller than the electrospinning fiber diameter, the electrospinning stack may be caused and was not able to be woven in the same plane. Therefore, the electrospinning pitch was determined to be ~10 μm, as shown in Figure 19b. Further analysis showed that the electrospun fibers were overlapped at a servo motor pitch of ~7 μm, as shown in Figure 19c. In order to confirm the performance of the developed servo motor electrospinning platform, a cross-laminated fiber was electrospun and could be mass-produced in the near future, as shown in Figure 19d.

The electrospun PVDF piezoelectric fiber by DW-NFES was covered with a dynamic sensor made of parallel electrodes and interdigitated electrodes with different pole pairs (10 and 15 pairs of parallel electrodes) for voltage measurement. The result showed that when the tapping test was done at a fixed frequency of 7 Hz, the parallel electrodes and the interdigitated electrodes with different pole pairs (10 and 15 pairs of parallel electrodes) are covered, the strain rate was ~7.80 × 10^−3^ s^−1^ and the maximum output voltage of parallel electrodes is ~38 mV. Furthermore, the maximum output voltage of 10 pole pairs of interdigitated electrodes is ~33 mV, and the maximum output voltage of 15 pole pairs of interdigitated electrodes is ~26 mV. Theoretically, the more the number of pole pairs, the higher the output voltage would be. However, as can be seen from the result that the number of pole pairs was inversely proportional to the output voltage, which may be due to polarity direction of the PVDF fibers between pair of positive and negative electrodes was canceled when the PVDF fibers were not repolarized, leading to the inability to generate a higher voltage output from the interdigitated electrode with multi-pole pairs. On the other hand, when the PVDF piezoelectric fiber was not applied in the polarization process, the longer the PVDF piezoelectric fiber, the higher the internal impedance, so the voltage output is greatly reduced. As a result, through the repolarization process, when the tapping test was done at a fixed frequency of 7 Hz, the parallel electrodes and the interdigitated electrodes with different pole pairs (10 and 15 pairs of parallel electrodes) are covered, the strain rate is ~7.80 × 10^−3^ s^−1^, the maximum output voltage of parallel electrodes is ~42 mV, the maximum output voltage of 10 pole pairs of interdigitated electrodes is ~85 mV, and the maximum output voltage of 15 pole pairs of interdigitated electrodes is ~125 mV. From the result above, it can be proved that repolarization can change the polarity direction of the PVDF fiber between each pair of positive and negative electrodes. The PVDF fiber between each pair of positive and negative electrodes can be regarded as a separate power generation unit. Each divided PVDF fiber power generation unit can achieve an additive effect by collecting the generated electric charge from the interdigitated electrode.

**Figure 20:**
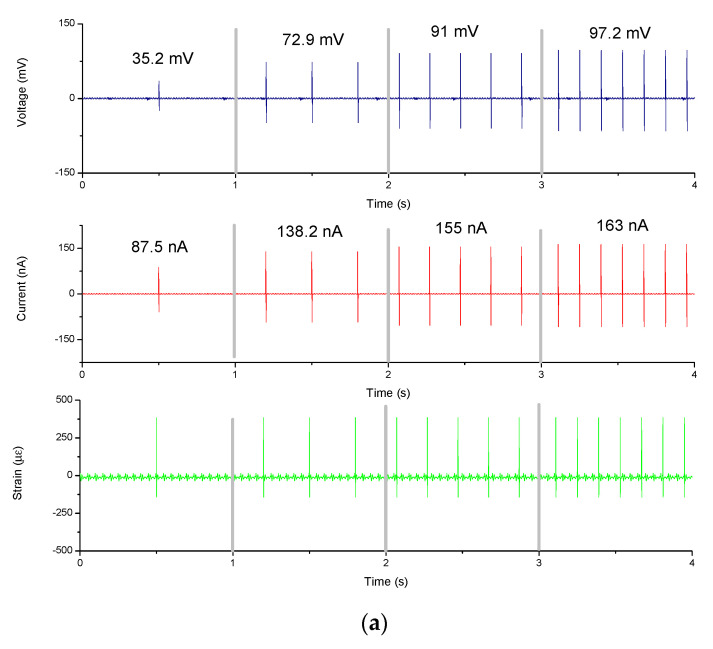

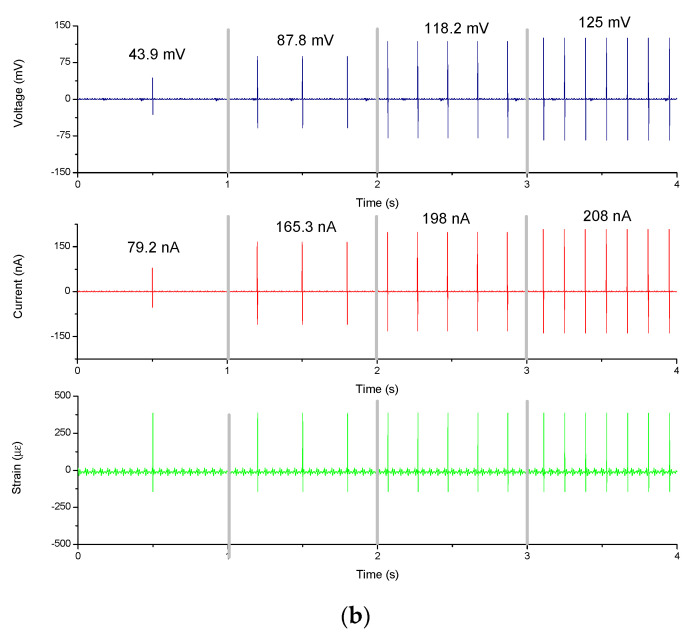
The electrical energy output results at different frequencies: (**a**) stepper motor electrospinning; (**b**) servo motor electrospinning.

**Figure 21:**
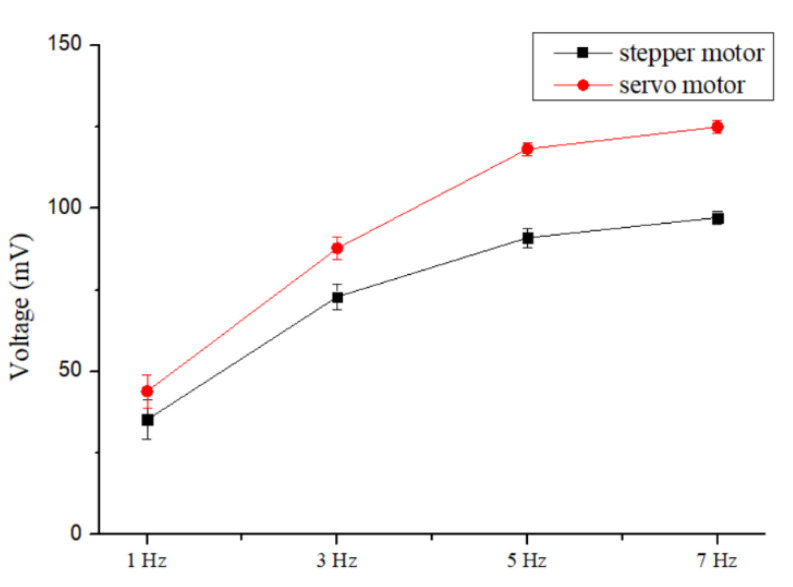
PVDF electrospinning voltage output value of tapping electrode at different frequencies in the stepper motor and the servo motor.

Figure 20 shows the electrical test which beat at 1, 3, 5, and 7 Hz, respectively. Overall, as the frequency of tapping increased, the electrical output (including voltage and current) elevated. It can be suggested that the amount of deformation was proportional to the electrical output of the electrospun fibers. It was found that the output voltage of the fibers of the stepping motor reached ~97.2 mV and the output current reached ~163 nA at a frequency of 7 Hz, as shown in Figure 20a. At 7 Hz, the output voltage of the fibers from the servo motor reached ~125 mV and the output current reached ~208 nA, as shown in Figure 20b. This may be explained by increase of number of fiber diameters within a single-unit area, next enhanced the electrical conductivity per unit area. According to the above results, the statistics of fiber power generation were shown in Figure 21. It was found that fibers generated by the servo motor generated a large amount of power at 7 Hz, from ~97.2 to ~125 mV. The current output was increased from ~163 to ~208 nA compared to the stepper motor.

**Figure 22:**
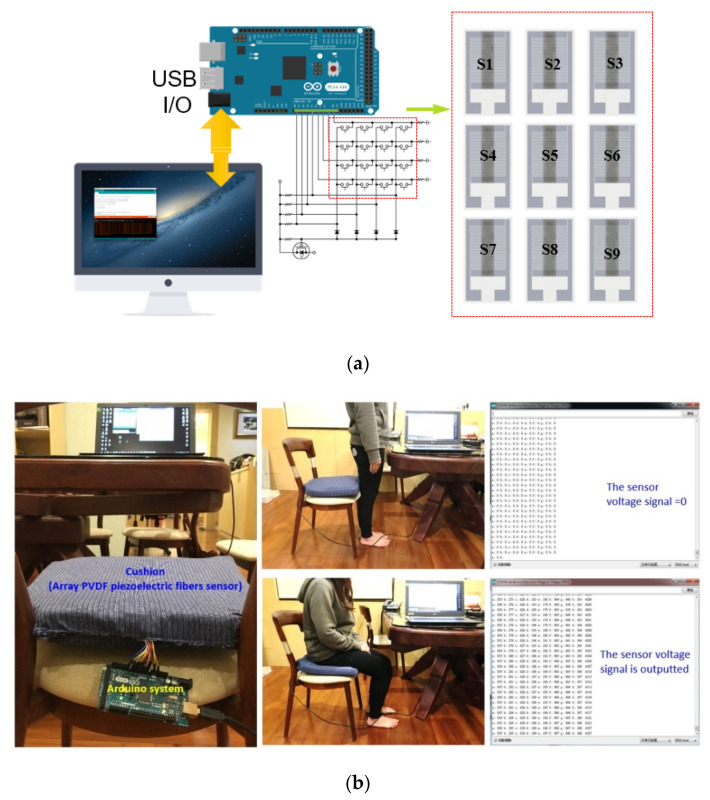
(**a**) Controller circuit model system (Arduino MAGE) and combining the sensing method of an array; (**b**) The patient standing voltage output signal was zero, the patient sitting appeared at a different voltage output signal.

The interdigitated electrodes with 15 repolarized pole pairs into 3 × 3 array sensing area was finally completed, and the sensing of large area for testers was also carried out. Further, the controller circuit model system (Arduino MAGE) combined with the sensing method of an array (as shown Figure 22a) was imported. The system successfully detected that the pressure of sedentary patients with the various values of the standing or sitting postures. Furthermore, when the cushion was subjected to different forces and positions, it revealed different electrical values. In this case, the accumulation system time can be monitored and used to determine whether the patients have to flip so as to avoid a fixed position for a long time, as shown Figure 22b.

## 4. Conclusions

In this study, the DW-NFES platform was developed with three-axis positioning of controllable speed, torque, and position to produce dense and high-quality piezoelectric fibers for the sensor purpose. The DW-NFES platform was retooled with a servo motor control. In the simulation result of the controller design, the current loop bandwidth was elevated from ~137 to ~437 Hz. The rise time was reduced from ~2.3 to ~0.69 ms. The speed loop bandwidth was increased from ~38 to ~57 Hz. The rise time decreased from ~6.9 to ~2.1 ms, and the overshoot decreased from ~51.5% to ~24.9%, where speed ripple notably reduced. The results of stepper and servo motor showed that the speed of this platform increased from ~20 s to ~125 mm/s and the precision reached from ~50 to ~1 μm. Further analysis showed that the electrospun fibers were overlapped at a servo motor pitch of ~7 μm. It was found that fibers generated by the servo motor generated a large amount of power at 7 Hz, from ~97.2 to ~125 mV. The current output was increased from ~163 to ~208 nA compared to the stepper motor. The interdigitated electrodes with 15 repolarized pole pairs in 3 × 3 array sensing area was completed for the large area sensing. The controller circuit model system (Arduino MAGE) combined with the sensing method of an array was incorporated. The system successfully detected the pressure of sedentary patients. When the cushion was subjected to different forces and positions, it showed different electrical values. The accumulation time can be used to determine whether or not the patient has to flip to avoid potential bedsores.

## Figures and Tables

**Table 1:** Technical data of silver paste PAX-767-2.

**Physical Properties**	
Color	Silver gray
Solid Content	About 79.1 wt%
Adhesion	100/100 (ITO Film)
Pencil Hardness	HB (ITO Film)
Resistivity	≤2 × 10^−4^ (Ω-cm)
Viscosity	145,000 ± 30,000 (cps)
Thixotropic Value (T.I)	≥4.8
Particle Size	≤3 μm
**Slurry Processing Data**	
Curing Conditions	Oven 130 °C/30 min
Scraper (angle; pressure; speed)	70 °C; 0.4–0.5 (MPa) 80–100 (mm/sec)
Screen Diameter	18 μm

**Table 2:** Stepper motor and servo motor comparison table.

	Stepper Motor	Servo Motor
Cost	Low	High
Control	Easy	Hard
Response	Slow	Fast
Speed Range	Low speed	High speed or low speed
Loop	Open-loop	Close-loop
Positioning Accuracy	Stable at low speed, high speed will lose step	Based on controller design

**Table 3:** Current loop step response parameter.

	Traditional Method	High Bandwidth
Rising Time	~2.3 (ms)	~0.69 (ms)
Settling Time	~8.9 (ms)	~4.4 (ms)
Overshoot	~9.3 (%)	~9.1 (%)
Peak Time	~5.2 (ms)	~2 (ms)

**Table 4:** Speed loop step response parameter.

	Traditional Method	High Bandwidth
Rise Time	~6.9 (ms)	~2.1 (ms)
Settling Time	~97.2 (ms)	~35.6 (ms)
Overshoot	~51.5 (%)	~24.9 (%)
Peak Time	~19.5 (ms)	~13.7 (ms)

**Table 5:** Stepper motor and servo motor comparison table.

	Stepper Motor	Servo Motor
Max Speed	20 mm/s	125 mm/s
Positioning Accuracy	50 μm	1 μm
Position: Rising Time	--	4 ms
Speed: Rising Time	--	34 ms
